# Eco Polymeric Materials and Natural Polymer

**DOI:** 10.3390/polym15194021

**Published:** 2023-10-08

**Authors:** Jingpeng Li, Yun Lu, Huiqing Wang

**Affiliations:** 1 Key Laboratory of High Efficient Processing of Bamboo of Zhejiang Province, China National Bamboo Research Center, Hangzhou 310012, China; 2 Research Institute of Wood Industry, Chinese Academy of Forestry, Beijing 100091, China; 3 Department of Polymer Science and Engineering, School of Chemical Engineering, Hefei University of Technology, Hefei 230009, China; huiqing.wang@hfut.edu.cn

With the increasing concern regarding the undesirable environmental and socioeconomic consequences of petrochemicals and limited fossil resources, biomass, bio-based polymers, and other renewable natural resources have increasingly become alternatives for the production of functional materials [1,2,3]. Natural biomasses, such as wood, bamboo, rattan, cellulose, bacterial cellulose, lignin, hemicellulose, chitin, alginate, silk, fibroin, starch, protein, collagen, gelatin, natural rubber, and their modified derivatives/composites, have been widely consumed for the preparation of bioplastics/biorubber in the form of films/members/hydrogels/foams/aerogels/fibers for various applications [4,5,6]. Biobased synthetic polymers such as polyester, poly(lactic acid) (PLA), polyhydroxyalkanoate (PHA), poly (butylene adipate-co-terephthalate) (PBAT), polycarbonate (PC), poly(butylene succinate) (PBS), polyurethane, and so on can be derived from a variety of molecular biomasses such as straw glucose, plant oils, fatty acids, furan, terpenes, rosin acids, and amino acids [7]. The use of such environmentally friendly or “green” polymer materials can avoid the dependence on petroleum resources and reduce carbon emissions [8,9]. Additionally, the green solvents, processes and technologies for polymers and the use of polymers for capturing pollution also contribute to the aim of global green and low-carbon transformation.

Nevertheless, eco polymeric materials and natural polymers face new challenges and problems every day. This Special Issue brings together different research works and reviews and attempts to cover the majority of the recent advances and applications of eco polymeric materials and natural polymers in the last few years.

This Special Issue gathers scientific works from research groups examining various eco polymeric materials, indicating advances in structural features, functions, and applications. The total number of manuscripts (30) published in this Special Issue indicates the importance of eco polymeric materials and natural polymers and the fact that many research groups and relevant members of the scientific community are thoroughly interested in the advancement of eco-friendly polymers and their advanced applications.

In their paper, Duan et al. (contribution 1) prepared an egg white dual cross-linked hydrogel through the induction of sodium hydroxide and the secondary cross-linking of protein chains by calcium ions. Characteristics of the dual cross-linked hydrogel were remarkably affected by the concentrations of calcium ions. The incorporation of calcium ions could benefit the thermal stability, swelling rate and texture of the hydrogels, while also reducing their swelling capacity. Calcium ions could impact the secondary structure of polypeptide chains and interact with protein chains, leading to more compact microstructure formation of the hydrogels. The results suggested that the egg white dual cross-linked hydrogels exhibited biocompatibility and cell-surface adhesion in vitro, indicating the potential for biomedical application.

In the study by Wu et al. (contribution 2), a stable composite hydrogel was prepared by incorporating konjac glucomannan (KGM) with oxidized hyaluronic acid (OHA), after which alkali processing and thermal treatment were conducted. The obtained hydrogel was pale yellow, smooth in surface, and had a favorable swelling capacity, which met the essential requirements for ideal drug-delivery applications. The OHA played an effective role in adjusting the swelling ratio and increasing the biodegradation rate. Furthermore, both the encapsulation efficiency of epigallocatechin gallate (EGCG) and the release properties of the hydrogels were significantly raised with the presence of OHA. The overall results suggest that the KGM/OHA hydrogel, loaded with EGCG, exhibited potential applications in controlled release.

In another study, Zhang et al. (contribution 3) prepared poly(vinyl alcohol)–chitosan/sodium alginate–Ca^2+^ (PVA–CS/SA–Ca^2+^) core–shell hydrogels with a bilayer space by cross-linking PVA and CS to form a core structure and chelating SA and Ca^2+^ to form a shell structure to achieve multiple substance loading and multifunctional expression. The SA concentration and SA/Ca^2+^ cross-linking time show a positive correlation with the thickness of the shell structure; the PVA/CS mass ratio affects the structural characteristics of the core structure; and a higher CS content indicates the more obvious three-dimensional network structure of the hydrogel. Their optimal experimental conditions for the swelling degree of the core–shell hydrogel included an SA concentration of 5%; an SA/Ca^2+^ cross-linking time of 90 min; a PVA/CS mass ratio of 1:0.7; and a maximum swelling degree of 50 g/g.

Another piece of research, conducted by Wei et al. (contribution 4), presented a facile and scalable method to produce a mass of chitosan tartaric ester via solvent-evaporation causing crystallization, in which tartaric acid was used as the crystallization and the crosslinking agent. In their article, chitosan tartaric sodium was prepared via hydrolysis with NaOH aqueous solution. As a result, the acquired nanostructured chitosan tartaric sodium, which is dispersed in an aqueous solution 20–50 nm in length and 10–15 nm in width, shows both the features of carboxyl and amino functional groups. Moreover, morphology regulation of the chitosan tartaric sodium nanostructures can be easily achieved by adjusting the solvent evaporation temperature. This work proves that this is a simple route to prepare chitosan-based nanostructure patterns.

A study by Filho et al. (contribution 5) found that the properties of PLA can be tailored by adding small concentrations of ethylene elastomeric grafted with glycidyl methacrylate (EE-g-GMA) and poly(ethylene-octene) grafted with glycidyl methacrylate (POE-g-GMA), generating promising eco-friendly materials. The blends PLA/EE-g-GMA and PLA/POE-g-GMA showed better impact properties and thermal stability compared to pure PLA. The increase in crystallinity contributed to maintaining the thermomechanical strength, Shore D hardness, and shifting the thermal stability of the PLA/EE-g-GMA and PLA/POE-g-GMA blends to a higher temperature. The obtained results suggested a good interaction between PLA and the EE-g-GMA and POE-g-GMA systems, due to the glycidyl methacrylate functional group. In light of this, new environmentally friendly and semi-biodegradable materials can be manufactured for application in the packaging industry.

In their paper, Sun et al. (contribution 6) prepared a carboxymethyl bacterial cellulose-based composite film with good thermal stability and mechanical properties. For the composite films with the addition of 1.5% carboxymethyl bacterial cellulose (% *v*/*v*), 1% sodium alginate, and 0.4% glycerin, the tensile strength was 38.13 MPa, the elongation at break was 13.4%, the kinematic viscosity of the film solution was 257.3 mm^2^/s, the opacity was 4.76 A/mm, the water vapor permeability was 11.85%, and the pyrolysis residue was 45%. Regression analysis of the data on mechanical properties yielded a significant correlation between thickeners and plasticizers regarding the tensile strength and elongation at break of the composite films.

In order to improve the survival rate of transplanted seedlings and improve the efficiency of seedling transplantation, Wang et al. (contribution 7) developed an environmentally friendly polymer konjac glucomannan (KGM)/chitosan (CA)/poly(vinyl alcohol) (PVA) ternary blend soil consolidation agent to consolidate the soil ball at the root of transplanted seedlings. They found that the film-forming performance of the adhesive was better when the KGM content was 4.5%, the CA content was in the range of 2–3%, the PVA content was in the range of 3–4%, and the preparation temperature was higher than 50 °C. The polymer soil consolidation agent prepared under this condition has good application prospects in seedling transplanting.

As one of the hazardous heavy metal ion pollutants, Cr(VI) has attracted much attention in the sewage treatment research field due to its broad distribution range and serious toxicity [10]. Wang et al. (contribution 8) developed a simple and effective strategy for preparing cellulose fibers with a stable 3D network structure using dissolution, regeneration, wet spinning, and freeze drying. Based on the rich pore structure of cellulose fibers, thioglycolic acid was used to deeply sulfhydryl-modify them to obtain sulfhydryl-modified cellulose fibers for efficient and rapid adsorption of Cr(VI). The maximum adsorption capacity of sulfhydryl-modified cellulose fibers to Cr(VI) can reach 120.60 mg g^−1^, the adsorption equilibrium can be achieved within 300 s, and its adsorption rate can reach 0.319 mg g^−1^ s^−1^. The in-depth sulfhydryl-modified cellulose fibers are also available for other heavy metal ions. The low cost and environmentally friendly properties of the as-synthesized material demonstrate its potential for practical usage for the treatment of heavy metal ion pollution in wastewater.

The paper by Yao et al. (contribution 9) investigated the effects of five plant sources on the resulting properties of sodium carboxymethyl cellulose (CMC) and CMC/sodium alginate/glycerol composite films. The degree of substitution and resulting tensile strength tended to be 20% lower in leaf-derived CMC compared to those prepared from wood or bamboo. Microstructures of bamboo cellulose, bamboo CMC powder, and bamboo leaf CMC composites’ films all differed from pine-derived material, but plant sources had no noticeable effect on the X-ray diffraction characteristics, Fourier transform infrared spectroscopy spectra, or pyrolysis properties of the CMC or composite films. The results highlighted the potential for using plant sources as a tool for varying CMC properties for specific applications.

In one study, Hakim et al. (contribution 10) examined the performance of citric acid-bonded orientation boards from a modified fibrovascular bundle salacca frond under NaOH + Na_2_SO_3_ treatment and the bonding mechanism between the modified fibrovascular bundle frond and citric acid. Their results found that the combination of 1% NaOH + 0.2% Na_2_SO_3_ treatment for 30 and 60 min immersion is successful in reducing the water absorption and thickness swelling of the orientation board. The findings of this study indicated that there is a reaction between the hydroxyl group in the modified fibrovascular bundle and the carboxyl group in citric acid.

A one-dimensional heat transfer model of natural fiber-reinforced thermoplastic composites during hot pressing was established by Qi et al. (contribution 11). The novelty of this study is that the apparent heat capacity of thermoplastics was first simulated and then coupled with the heat transfer model to simulate the temperature distribution of natural fiber-reinforced thermoplastics composites during hot pressing. Both the experimental and simulated data suggested that a higher temperature and/or a longer duration during the hot-pressing process should be used to fabricate oriented sorghum fiber-reinforced high-density polyethylene film composites as the high-density polyethylene content increases.

Another interesting paper by Shahzad et al. (contribution 12) evaluated the environmental burden of polyhydroxyalkanoate production from slaughtering residues by utilizing the Emergy Accounting methodology. The emergy intensity for polyhydroxyalkanoate production (seJ/g) shows a minor improvement ranging from 1.5% to 2% by changing only the electricity provision resources. This impact reaches up to 17% when electricity and heat provision resources are replaced with biomass resources. Similarly, the emergy intensity for polyhydroxyalkanoate production using electricity EU27 mix, coal, hydropower, wind power, and biomass is about 5% to 7% lower than the emergy intensity of polyethylene high density. In comparison, its value is up to 21% lower for electricity and heat provision from biomass.

Wood is a typical natural polymeric material. Wood drying is an essential step in wood processing, and it is also the most energy- and time-demanding step. Fu et al. (contribution 13) presented an electrochemical method to determine wood moisture content and shrinkage strain during drying. As the moisture content changed from 42% to 12%, the resistance increased from 1.0 × 10^7^ Ω to 1.2 × 10^8^ Ω. Both the shrinkage strain and resistance change rate increased with the decrease in wood moisture content, especially for the moisture content range of 23% to 8%, where the shrinkage strain and resistance change rate increased by 4% and 30%, respectively. This demonstrated the feasibility of the electrochemical method for measuring wood moisture content and shrinkage strain.

Wood modification can improve the dimensional stability, strength, and other properties of wood, and it has been extensively used. Hu et al. (contribution 14) improved the dimensional stability of wood via the in situ polymerization of water-soluble monomers in water. 2-Hydroxyethyl methacrylate and glyoxal were injected into the wood cell walls and activated cross-linking reactions to form interpenetrating polymer network structures. The polymer network blocked the partial pores and reduced wood hydroxyl, which simultaneously and significantly increased the wood’s transverse connections, dimensions, and stability. This work advances fast-growing-wood modification by introducing a novel research strategy.

In another study, poplar veneer–thermoplastic composites and oriented strand–thermoplastic composites were fabricated by Shen et al. (contribution 15) using hot pressing. Their result found that the use of both KH550 and MDI as coupling agents improved the interfacial bond strength between wood and thermoplastics under dry conditions. The use of MDI resulted in a much greater increase in the interfacial bond strength than KH550 under both dry and wet conditions, while KH550 had a negative effect under wet conditions. The better interfacial bond strength between wood and thermoplastics provided oriented strand–thermoplastic composites with better mechanical properties and dimensional stability. The obtained results will guide the industry to produce high-performance wood–plastic composites using hot pressing for general applications.

During outdoor use, wood composites are susceptible to destruction by rot fungi. Bao et al. (contribution 16) investigated the effects of resin content and density on the resistance of outdoor wood mat-based engineering composites to fungal decay through fungal decay tests for a period of 12 weeks. The highest antifungal effects against *T. versicolor* (12.34% mass loss) and *G. trabeum* (19.43% mass loss) were observed at a density of 1.15 g/m^3^ and a resin content of 13%. As a result of the chemical composition and microstructure measurements, the resistance of the outdoor wood mat-based engineering composite against *T. versicolor* and *G. trabeum* fungi was improved remarkably by increasing the density and resin content. The results of this study will provide a technical basis to improve the decay resistance of wood mat-based engineering composite in outdoor environments.

In the study by Ye et al. (contribution 17), the stability coefficient calculation theories in different national standards were analyzed and then the stability bearing capacity of cross-laminated timber elements with four slenderness ratios was investigated. Their results show that the average deviation between the fitting curve and calculated results of European and American standard was 5.43% and 3.73%, respectively, and the average deviation between the fitting curve and the actual test results was 8.15%. The stability coefficients calculation formulae could be used to reliably predict the stability coefficients of cross-laminated timber specimens with different slenderness ratios.

Another interesting paper by Nun-Anan et al. (contribution 18) investigated the effects of Aquilaria crassna wood (ACW) on the antifungal, physical and mechanical properties of natural rubber as air-dried sheets (ADS) and ADS filled with ACW. They found that the ACW-filled ADS had an increased Mooney viscosity, initial plasticity, and high thermo-oxidation plasticity (i.e., high plasticity retention index PRI). Additionally, superior green strength was observed for the ACW-filled ADS over the ADS without an additive because of chemical interactions between lignin and proteins in the natural rubber molecules eliciting greater gel formation. A significant inhibition of fungal growth on the natural rubber products during storage over a long period (5 months) was observed for ACW-filled ADS. The results suggested that these filled intermediate natural rubber products provide added value through an environmentally friendly approach, which is attractive to consumers.

Bamboo is a natural fiber-reinforced composite with excellent performance which is, to a certain extent, an alternative to the shortage of wood resources. In one study, the distribution of lignin components and lignin content in bamboo micro-morphological regions was measured by Liu et al. (contribution 19) at a semi-quantitative level according to age and radial location by means of visible-light microspectrophotometry coupled with the Wiesner and Maule reaction. They found that lignification develops with aging. Guaiacyl lignin units and syringyl lignin units were found in the cell wall of the fiber, parenchyma, and vessel. Differences in lignin content among different ages, different radial locations, and different micro-morphological regions of the cell wall were observed in this paper. It is considered that lignin plays an important role in cell-wall formation and the cell wall’s mechanical properties. Lignin is related to the physical and mechanical properties of bamboo. Therefore, this study of the distribution of and change in lignin in bamboo development is conducive to the mastery and prediction of various properties in the bamboo development, and has guiding significance for bamboo and lignin industrial utilization.

In another study, combined microscopic techniques were used by Jin et al. (contribution 20) to non-destructively investigate the compositional heterogeneity and variation in cell wall mechanics in moso bamboo. Along the radius of bamboo culms, the concentration of xylan within the fiber sheath increased, while that of cellulose and lignin decreased gradually. At the cellular level, although the consecutive broad layer of fiber revealed a relatively uniform cellulose orientation and concentration, the outer broad layer with a higher lignification level has a higher elastic modulus (19.59–20.31 GPa) than that of the inner broad layer close to the lumen area (17.07–19.99 GPa). Comparatively, the cell corner displayed the highest lignification level, while its hardness and modulus were lower than that of the fiber broad layer, indicating that the cellulose skeleton is the prerequisite of cell-wall mechanics. The obtained cytological information is helpful to understand the origin of the anisotropic mechanical properties of bamboo.

Huang et al. (contribution 21) investigated the different shear performances of bamboo using four test methods: the tensile-shear, step-shear, cross-shear, and short-beam-shear methods. They indicated that the shear strength was significantly different in the four test methods and was highest in the step-shear-test method, but lowest in the tensile-shear-test method. The compound mode of compression and shear for the axial resulted in the maximum shear strength in the step-shear test, while the interface shear caused the tensile-shear strength to be the lowest. However, the shear changed the original fracture behavior of the tension, bending, and compression. Additionally, the axial-shear-test method caused typical interface-shear failure in the tensile-shear test and the overall tearing of fiber bundles in the step-shear test, while the parenchyma-cells collapsed in the cross-shear test. However, the short-beam-shear shearing characteristics resembled bending with the fiber bundle being pulled out. The findings of this study will inform the good use and manufacturing process of bamboo culm.

A study by Tang et al. (contribution 22) investigated the effects of tung oil thermal treatment on bamboo color at different temperatures and durations of time. The obtained results showed that the lightness (L*) of bamboo decreased as the tung oil temperature or duration of time increased. The red–green coordinates (a*) and color saturation (C*) of bamboo were gradually increased as the tung oil temperature rose from 23 °C to 160 °C, while the a* and C* were gradually decreased when the temperature continued to rise from 160 °C to 200 °C. Eye movement data showed that the popularity of bamboo furniture was significantly improved at 23–100 °C and slightly improved at 160–180 °C with tung oil treatment. The findings of this study suggested that tung oil thermal treatment played a positive role in improving the visual effects and additional value of bamboo.

In another study, natural resin rosin was used by Su et al. (contribution 23) to treat round bamboo culm using the impregnation method. The obtained results showed that proper heating of the modified system was conducive to the formation of a continuous rosin film, which increased the gloss value. Heating decreased the brightness of the bamboo culm and changed the color from the green and yellow tones to red and blue. However, the heating temperature should not exceed 60 °C. They also used eye tracking technology to evaluate the users’ preference for the visual characteristics of the bamboo culm surface. The findings of this study indicated that natural rosin resin could effectively improve the visual characteristics of bamboo culm, and different visual effects on bamboo culm surfaces were obtained in different temperature ranges.

Bamboo is easily attacked by fungus, resulting in a shorter service life and higher loss in storage and transportation [11]. The mold resistance of bamboo strips treated with low-molecular-weight organic acids and inorganic acid was first tested by Yu et al. (contribution 24), and then effect of citric acid with different concentrations was studied. Bamboo treated with acetic acid, propionic acid, oxalic acid, citric acid, and hydrochloric acid in a low concentration could improve their fungus growth rating from 4 in control samples to 2 or 3. Citric acid is effective in preventing mildew, and the mold resistance increased with the increased concentration of citric acid, and the fungus growth rating could reach 1 when the citric acid concentration was greater than 8%, while treating bamboo with citric acid in the concentration of 10% could control the infected area in the range of 10–17%. The improved mold and blue-stain resistance of treated bamboo could be attributed to the reduced nutrients in bamboo due to the hydrolysis of starch grains in parenchyma cells and the dissolution of soluble sugar.

In another paper, Peng et al. (contribution 25) synthesized the sustained-release system loading citral by using PNIPAm nanohydrogel as a carrier and analyzed its drug-release kinetics and mechanism. Their experimental results revealed that the release kinetics equation of the system conformed to the first order; the higher the external temperature, the better the match was. In the release process, PNIPAm demonstrated a good protection and sustained-release effect on citral. The laboratory mold control experiment results revealed that under the optimal conditions of release and impregnation time, the control efficiency of the bamboo treatment with pressure impregnation against the common bamboo molds, such as *P. citrinum*, *T. viride*, *A. niger*, and mixed mold reached 100% after 28 days, and the original colour of the bamboo was maintained during the mold control process.

Two manuscripts focusing on bamboo scrimber composites are also included in this Special Issue. In the first study, Ji et al. (contribution 26) prepared the bamboo scrimber composites using moso bamboo and phenol-formaldehyde resin, and the changes in the macroscopic and microscopic bonding interfaces before and after 28 h water-resistance tests were observed and analyzed. They showed that the water resistance of the bamboo scrimber composite increased with increasing resin content, with higher thickness swelling rates observed at higher densities. Obvious cracks were found at the macroscopic interface after 28 h tests, with higher resin contents leading to fewer and smaller cracks. With increasing density, the longitudinal fissures due to the defibering process decreased, having an effect on the width swelling rates. They suggested that the macroscopic and microscopic bonding interface structures of the bamboo scrimber composite are closely related to their water resistance.

The second study, by Wang et al. (contribution 27), investigated the influence of grain direction on the compression properties and failure mechanism of bamboo scrimber. They showed that the compressive load–displacement curves of bamboo scrimber in the longitudinal, tangential and radial directions contained elastic, yield and failure stages. The compressive strength and elastic modulus of the bamboo scrimber in the longitudinal direction were greater than those in the radial and tangential directions, and there were no significant differences between the radial and tangential specimens. The main failure mode of bamboo scrimber under longitudinal and radial compression was shear failure, and the main failure mode under tangential compression was interlayer separation failure. This study can provide benefits for the rational design and safe application of bamboo scrimber in practical engineering.

Moreover, Liang et al. (contribution 28) developed a facile strategy using the surfactant-induced reconfiguration of urea–formaldehyde (UF) resins to enhance the interface with bamboo and significantly improve its gluability. Through the coupling of a variety of surfactants, the viscosity and surface tension of the UF resins were properly regulated. The resultant surfactant-reconfigured UF resin showed much improved wettability and spreading performance to the surface of both green bamboo and yellow bamboo. Moreover, their reconfigured UF resin can reduce the amount of glue spread applied to bond the laminated commercial bamboo veneer products to 60 g m^−2^, while the products prepared using the initial UF resin are unable to meet the requirements of the test standard, suggesting that this facile method is an effective way to decrease the application of petroleum-based resins and production costs.

In addition, in another study, bamboo delignification is a common method for studying its functional value-added applications. In Yu et al. (contribution 29)’s study, bamboo samples were delignified by means of treatment with sodium chlorite. They demonstrated that the lignin peak decreased or disappeared, and some hemicellulose peaks decreased, indicating that sodium chlorite treatment effectively removed lignin and partly decomposed hemicellulose, although cellulose was less affected. They suggested that delignified bamboo develops loose surfaces, increased pores, and noticeable fibers, indicating that alkali-treated bamboo has promising application potential due to its novel and specific functionalities.

Finally, in their review article, Li et al. (contribution 30) summarized the methods for preparing transparent bamboo, including delignification and resin impregnation. Potential applications of transparent bamboo are discussed using various functionalizations achieved through doping nanomaterials or modified resins to realize advanced energy-efficient building materials, decorative elements, and optoelectronic devices. Finally, challenges associated with the preparation, performance improvement, and production scaling of transparent bamboo are summarized, suggesting opportunities for the future development of this novel, bio-based, and advanced material.

This Special Issue has brought together experts that have studied and explored various aspects of eco polymeric materials and natural polymers. We would like to thank all researchers who have contributed to the production of this Special Issue of *Polymers*. In addition, I would like to express my gratitude to the Editorial Team who helped prepare the “Eco Polymeric Materials and Natural Polymer” Special Issue.

## List of Contributions

1. Duan, B.; Yang, M.; Chao, Q.; Wang, L.; Zhang, L.; Gou, M.; Li, Y.; Liu, C.; Lu, K., Preparation and Properties of Egg White Dual Cross-Linked Hydrogel with Potential Application for Bone Tissue Engineering. *Polymers* 2022, 14(23), 5116; https://doi.org/10.3390/polym14235116.
2. Wu, H.; Bu, N.; Chen, J.; Chen, Y.; Sun, R.; Wu, C.; Pang, J., Construction of Konjac Glucomannan/Oxidized Hyaluronic Acid Hydrogels for Controlled Drug Release. *Polymers* 2022, 14(5), 927; https://doi.org/10.3390/polym14050927.
3. Zhang, S.; Wan, Y.; Yuan, W.; Zhang, Y.; Zhou, Z.; Zhang, M.; Wang, L.; Wang, R., Preparation of PVA–CS/SA–Ca^2+^ Hydrogel with Core–Shell Structure. *Polymers* 2022, 14(1), 212; https://doi.org/10.3390/polym14010212.
4. Wei, S.; Peng, R.; Bian, S.; Han, W.; Xiao, B.; Peng, X., Facile and Scalable Synthesis and Self-Assembly of Chitosan Tartaric Sodium. *Polymers* 2022, 14(1), 69; https://doi.org/10.3390/polym14010069.
5. dos Santos Filho, E. A.; Luna, C. B. B.; Siqueira, D. D.; Barbosa Ferreira, E. d. S.; Araujo, E. M., Tailoring Poly(lactic acid) (PLA) Properties: Effect of the Impact Modifiers EE-g-GMA and POE-g-GMA. *Polymers* 2022, 14(1), 136; https://doi.org/10.3390/polym14010136.
6. Sun, Z.; Tang, Z.; Li, X.; Li, X.; Morrell, J. J.; Beaugrand, J.; Yao, Y.; Zheng, Q., The Improved Properties of Carboxymethyl Bacterial Cellulose Films with Thickening and Plasticizing. *Polymers* 2022, 14(16), 3286; https://doi.org/10.3390/polym14163286.
7. Wang, S.; Song, S.; Yang, X.; Xiong, Z.; Luo, C.; Xia, Y.; Wei, D.; Wang, S.; Liu, L.; Wang, H.; Sun, L.; Du, L.; Li, S., Effect of Preparation Conditions on Application Properties of Environment Friendly Polymer Soil Consolidation Agent. *Polymers* 2022, 14(10), 2122; https://doi.org/10.3390/polym14102122.
8. Wang, W.; Yu, F.; Ba, Z.; Qian, H.; Zhao, S.; Liu, J.; Jiang, W.; Li, J.; Liang, D., In-Depth Sulfhydryl-Modified Cellulose Fibers for Efficient and Rapid Adsorption of Cr(VI). *Polymers* 2022, 14(7), 1482; https://doi.org/10.3390/polym14071482.
9. Yao, Y.; Sun, Z.; Li, X.; Tang, Z.; Li, X.; Morrell, J. J.; Liu, Y.; Li, C.; Luo, Z. J. P., Effects of Raw Material Source on the Properties of CMC Composite Films. *Polymers* 2022, 14(1), 32; https://doi.org/10.3390/polym14010032.
10. Hakim, L.; Widyorini, R.; Nugroho, W. D.; Prayitno, T. A., Performance of Citric Acid-Bonded Oriented Board from Modified Fibrovascular Bundle of Salacca (*Salacca zalacca* (Gaertn.) Voss) Frond. *Polymers* 2021, 13(23), 4090; https://doi.org/10.3390/polym13234090.
11. Qi, C.; Wang, J.; Yadama, V., Heat Transfer Modeling of Oriented Sorghum Fibers Reinforced High-Density Polyethylene Film Composites during Hot-Pressing. *Polymers* 2021, 13(21), 3631; https://doi.org/10.3390/polym13213631.
12. Shahzad, K.; Rehan, M.; Rashid, M. I.; Ali, N.; Summan, A. S.; Ismail, I. M. I., Sustainability Evaluation of Polyhydroxyalkanoate Production from Slaughterhouse Residues Utilising Emergy Accounting. *Polymers* 2022, 14(1), 118; https://doi.org/10.3390/polym14010118.
13. Fu, Z.; Wang, H.; Li, J.; Lu, Y., Determination of Moisture Content and Shrinkage Strain during Wood Water Loss with Electrochemical Method. *Polymers* 2022, 14(4), 778; https://doi.org/10.3390/polym14040778.
14. Hu, J.; Fu, Z.; Wang, X.; Chai, Y., Manufacturing and Characterization of Modified Wood with In Situ Polymerization and Cross-Linking of Water-Soluble Monomers on Wood Cell Walls. *Polymers* 2022, 14(16), 3299; https://doi.org/10.3390/polym14163299.
15. Shen, Z.; Ye, Z.; Li, K.; Qi, C., Effects of Coupling Agent and Thermoplastic on the Interfacial Bond Strength and the Mechanical Properties of Oriented Wood Strand–Thermoplastic Composites. *Polymers* 2021, 13(23), 4260; https://doi.org/10.3390/polym13234260.
16. Bao, M.; Li, N.; Bao, Y.; Li, J.; Zhong, H.; Chen, Y.; Yu, Y., Outdoor Wood Mats-Based Engineering Composite: Influence of Process Parameters on Decay Resistance against Wood-Degrading Fungi *Trametes versicolor* and *Gloeophyllum trabeum*. *Polymers* 2021, 13(18), 3173; https://doi.org/10.3390/polym13183173.
17. Ye, Q.; Gong, Y.; Ren, H.; Guan, C.; Wu, G.; Chen, X., Analysis and Calculation of Stability Coefficients of Cross-Laminated Timber Axial Compression Member. *Polymers* 2021, 13(23), 4267; https://doi.org/10.3390/polym13234267.
18. Nun-Anan, P.; Suchat, S.; Mahathaninwong, N.; Chueangchayaphan, N.; Karrila, S.; Limhengha, S., Study of *Aquilaria crassna* Wood as an Antifungal Additive to Improve the Properties of Natural Rubber as Air-Dried Sheets. *Polymers* 2021, 13(23), 4178; https://doi.org/10.3390/polym13234178.
19. Liu, B.; Tang, L.; Chen, Q.; Zhu, L.; Zou, X.; Li, B.; Zhou, Q.; Fu, Y.; Lu, Y., Lignin Distribution on Cell Wall Micro-Morphological Regions of Fibre in Developmental *Phyllostachys pubescens* Culms. *Polymers* 2022, 14(2), 312; https://doi.org/10.3390/polym14020312.
20. Jin, K.; Ling, Z.; Jin, Z.; Ma, J.; Yang, S.; Liu, X.; Jiang, Z., Local Variations in Carbohydrates and Matrix Lignin in Mechanically Graded Bamboo Culms. *Polymers* 2022, 14(1), 143; https://doi.org/10.3390/polym14010143.
21. Huang, A.; Su, Q.; Zong, Y.; Chen, X.; Liu, H., Study on Different Shear Performance of Moso Bamboo in Four Test Methods. *Polymers* 2022, 14(13), 2649; https://doi.org/10.3390/polym14132649.
22. Tang, T.; Fei, B.; Song, W.; Su, N.; Sun, F., Tung Oil Thermal Treatment Improves the Visual Effects of Moso Bamboo Materials. *Polymers* 2022, 14(6), 1250; https://doi.org/10.3390/polym14061250.
23. Su, N.; Fang, C.; Zhou, H.; Tang, T.; Zhang, S.; Wang, X.; Fei, B., Effect of Rosin Modification on the Visual Characteristics of Round Bamboo Culm. *Polymers* 2021, 13(20), 3500; https://doi.org/10.3390/polym13203500.
24. Yu, Z.; Zhang, X.; Zhang, R.; Yu, Y.; Sun, F., Improving the Mould and Blue-Stain-Resistance of Bamboo through Acidic Hydrolysis. *Polymers* 2022, 14(2), 244; https://doi.org/10.3390/polym14020244.
25. Peng, R.; Zhang, J.; Du, C.; Li, Q.; Hu, A.; Liu, C.; Chen, S.; Shan, Y.; Yin, W., Investigation of the Release Mechanism and Mould Resistance of Citral-Loaded Bamboo Strips. *Polymers* 2021, 13(19), 3314; https://doi.org/10.3390/polym13193314.
26. Ji, Y.; Lei, W.; Huang, Y.; Wu, J.; Yu, W., Influence of Resin Content and Density on Water Resistance of Bamboo Scrimber Composite from a Bonding Interface Structure Perspective. *Polymers* 2022, 14(9), 1856; https://doi.org/10.3390/polym14091856.
27. Wang, X. Y.; Zhong, Y.; Luo, X. Y.; Ren, H. Q., Compressive Failure Mechanism of Structural Bamboo Scrimber. *Polymers* 2021, 13(23), 4223; https://doi.org/10.3390/polym13234223.
28. Liang, L.; Zheng, Y.; Wu, Y.; Yang, J.; Wang, J.; Tao, Y.; Li, L.; Ma, C.; Pang, Y.; Chen, H.; Yu, H.; Shen, Z., Surfactant-Induced Reconfiguration of Urea-Formaldehyde Resins Enables Improved Surface Properties and Gluability of Bamboo. *Polymers* 2021, 13(20), 3542; https://doi.org/10.3390/polym13203542.
29. Yu, H.; Gui, C.; Ji, Y.; Li, X.; Rao, F.; Huan, W.; Li, L., Changes in Chemical and Thermal Properties of Bamboo after Delignification Treatment. *Polymers* 2022, 14(13), 2573; https://doi.org/10.3390/polym14132573.
30. Li, X. L.; Zhang, W. Z.; Li, J. P.; Li, X. Y.; Li, N.; Zhang, Z. H.; Zhang, D. P.; Rao, F.; Chen, Y. H., Optically Transparent Bamboo: Preparation, Properties, and Applications. *Polymers* 2022, 14(16), 3234; https://doi.org/10.3390/polym14163234.

## References

[1] Cywar R.M., Rorrer N.A., Hoyt C.B., Beckham G.T., Chen E.Y.X. (2022). Bio-based polymers with performance-advantaged properties. Nat. Rev. Mater..

[2] Garrison T.F., Murawski A., Quirino R.L. (2016). Bio-Based Polymers with Potential for Biodegradability. Polymers.

[3] Li J., Ma R., Yao S., Qin D., Lu Y., Chen Y., Jiang Z., Yang D. (2023). Ultrafine Pd Nanoparticles Encapsulated in Mesoporous TiO_2_ Region Selectively Confined in Bamboo Microchannels: An Ultrastable Continuous-Flow Catalytic Hydrogenation Microreactor. Small Struct..

[4] Brodin M., Vallejos M., Opedal M.T., Area M.C., Chinga-Carrasco G. (2017). Lignocellulosics as sustainable resources for production of bioplastics–A review. J. Clean. Prod..

[5] Nandakumar A., Chuah J.-A., Sudesh K. (2021). Bioplastics: A boon or bane?. Renew. Sustain. Energy Rev..

[6] Sun S., Zhang J., Zhang L., Liu S., Wang R., Kang H., Wang Z. (2013). Research Progress in Biorubber. Polym. Bull..

[7] Phadke G., Rawtani D. (2023). Bioplastics as polymeric building blocks: Paving the way for greener and cleaner environment. Eur. Polym. J..

[8] Muelhaupt R. (2013). Green Polymer Chemistry and Bio-based Plastics: Dreams and Reality. Macromol. Chem. Phys..

[9] Zhao H., Wang J., Meng Y., Li Z., Fei B., Das M., Jiang Z. (2022). Bamboo and rattan: Nature-based solutions for sustainable development. Innovation.

[10] Mallik A.K., Moktadir M.A., Rahman M.A., Shahruzzaman M., Rahman M.M. (2022). Progress in surface-modified silicas for Cr(VI) adsorption: A review. J. Hazard. Mater..

[11] Yan J., Niu Y., Wu C., Shi Z., Zhao P., Naik N., Mai X., Yuan B. (2021). Antifungal effect of seven essential oils on bamboo. Adv. Compos. Hybrid Mater..

